# The Complete Chloroplast Genomic Characteristics and Phylogenetic Analysis of *Abutilon theophrasti* Medicus

**DOI:** 10.3390/ijms27031205

**Published:** 2026-01-25

**Authors:** Changli Chen, Xiahong Luo, Ziyi Zhu, Xingcai An, Junyuan Dong, Qingqing Ji, Tingting Liu, Lina Zou, Shaocui Li, Jikang Chen, Xia An

**Affiliations:** 1 Zhejiang Xiaoshan Institute of Cotton & Bast Fiber Crops, Zhejiang Institute of Landscape Plants and Flowers, Zhejiang Academy of Agricultural Sciences, Hangzhou 311251, China; chenchangli@zaas.ac.cn (C.C.); luoxh@zaas.ac.cn (X.L.); 13456319193@163.com (Z.Z.); xcan2001str@163.com (X.A.); 13964552682@163.com (J.D.); jiqingqing1001@163.com (Q.J.); liutt@zaas.ac.cn (T.L.); zoulina1991@yeah.net (L.Z.); lishaocui@zaas.ac.cn (S.L.); 2 College of Environment and Resources, College of Carbon Neutrality, Zhejiang A&F University, Hangzhou 311300, China; 3 School of Agriculture, Yunnan University, Kunming 650500, China; 4 Institute of Bast Fiber Crops, Chinese Academy of Agricultural Sciences/Key Laboratory of Bast Fiber Biology and Processing, Ministry of Agriculture and Rural Affairs, Changsha 410221, China

**Keywords:** *Abutilon theophrasti* Medicus, chloroplast genome, feature analysis, phylogeny

## Abstract

To clarify the phylogenetic relationship between *Abutilon theophrasti* M. and other *Malvaceae* plants, the chloroplast genome of *A. theophrasti* was assembled, annotated, and analyzed. The complete chloroplast genome was sequenced using the Illumina NovaSeq 6000 platform. Bioinformatics methods were employed to systematically analyze its genomic structure, repetitive sequences, nucleic acid diversity, and codon preference. Additionally, a phylogenetic tree was constructed by integrating chloroplast genomic sequences from other *Malvaceae* species. The results showed that the chloroplast genome of *A. theophrasti* was 160,440 bp in length with a GC content of 36.89%, exhibiting a typical tetrad structure. A total of 130 coding genes were annotated, including 85 mRNA genes, 37 tRNA genes, and 8 rRNA genes, with no pseudogenes detected. Codon preference analysis indicates that leucine (Leu) is the most frequently used amino acid. There are 31 codons with a relative synonymous codon usage (RSCU) value greater than 1, most of which end with A or U. The genome contains 61 scattered repeat sequences and 288 simple repeat sequences (SSR). Ka/Ks analysis revealed that the overall chloroplast genes of *A. theophrasti* undergo purifying selection, while genes such as *psbK* and *rps12* are subjected to positive selection, which may be associated with adaptive evolution. Phylogenetically, *A. theophrasti* is most closely related to its congener *A. indicum*, followed by a clade comprising *M. cathayensis* and *Malva crispa* of the genus *Malva*. This study enhances the understanding of the phylogenetic relationship of *A. theophrasti* and provides a theoretical basis for the genetic improvement and breeding strategies of *A. theophrasti* and other *Malvaceae* plants.

## 1. Introduction

*Abutilon theophrasti* Medicus, commonly known as green hemp or white hemp, etc., is an annual herb native to China and widely distributed in temperate and tropical regions [1]. Historically, it has been valued for medicinal and ornamental purposes, as well as a source of fiber and oil crop [2], boasting substantial economic and medicinal potential. The stem bark fibers of *A*. *theophrasti* are tough, making it a novel natural textile material [3,4]. The entire plant and seeds are used in traditional Chinese medicine, characterized by a bitter taste and neutral nature, with effects of clearing heat, promoting diuresis, detoxifying, and removing cataracts. It is frequently employed to treat dysentery, abscesses, cataracts, and other conditions [5]. Modern research has identified active components such as flavonoids and alkaloids in *A. theophrasti,* which exhibit anti-inflammatory and antioxidant properties, though its specific pharmacological mechanism requires further investigation [6].

Plant chloroplasts are semi-autonomous genetic organelles with a double-membrane structure [7]. By converting light energy into ATP and carbohydrate energy, they directly determine crop yields. Adverse environmental factors such as drought, flood, salinity, extreme temperature, nutritional imbalance, and pathogen/virus infections can disrupt photosynthetic function, leading to significant yield reduction. Chloroplasts possess three types of membranes: inner, outer, and thylakoid membranes, each equipped with specific ion channels and transport proteins that efficiently mediate the transmembrane transport of nutrients, solutes, and metabolites [8]. They play crucial roles in processes such as photosynthesis and carbohydrate metabolism [9]. In recent years, advances in high-throughput technology have deepened research on plant chloroplast genomes. The majority of higher plants have a typical tetrameric chloroplast genome structure consisting of a large single copy region (LSC), a small single copy region (SSC), and two inverted repeat regions (A and B). IRA and IRB consist of four parts [10,11]. The chloroplast genome is maternally inherited and highly conserved, unaffected by the nuclear genome. These characteristics make chloroplast genomes ideal for studies on plant phylogeny and genetic diversity analysis [12,13], with significant applications in the research of plant system evolution, the analysis of photosynthetic molecular mechanisms, and genetic engineering [14].

The *Malvaceae* family is rich in species, exhibiting considerable morphological and ecological diversity. As a medium-to-large family in the plant kingdom, it holds significant ecological and economic importance [15,16]. *A*. *theophrasti* belongs to the *Malvaceae* subfamily, which is the most species-rich and representative core subfamily of *Malvaceae* [17], and is one of its economically and medicinally valuable species. However, the phylogenetic position of *A. theophrasti* within the *Malvaceae* family and its evolutionary relationship with related species remain unclear. Chloroplast genomes can provide abundant genetic information for plant evolution studies. In this study, *A. theophrasti* was used as the experimental material, and high-throughput sequencing technology and bioinformatics methods were employed to sequence, assemble, and annotate its chloroplast genome, followed by in-depth analysis of its structural characteristics and functional genes. Additionally, phylogenetic analysis was conducted to clarify the evolutionary position of *A. theophrasti* in the *Malvaceae*. The results provide a theoretical foundation for the phylogenetic study of *Malvaceae*, as well as the protection and utilization of germplasm resources.

## 2. Results

### 2.1. Basic Characteristics of A. theophrasti M. chloroplast Genome

**Figure 1:**
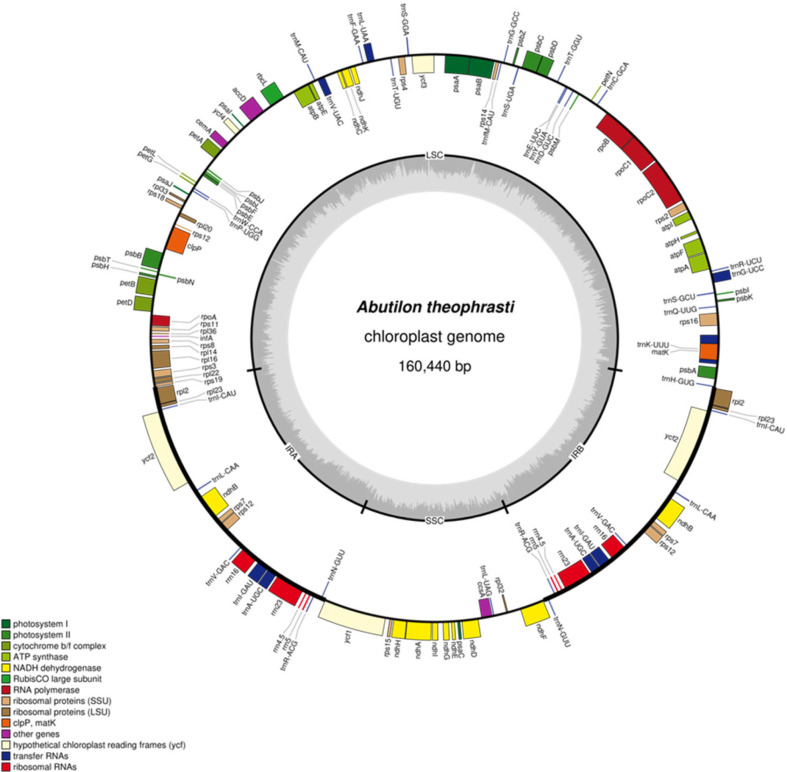
Chloroplast genome map of *A. theophrasti*. Note: Genes encoded in the forward direction are located on the outside of the circle, while genes encoded in the reverse direction are located on the inside of the circle. The inner gray circle represents the GC content.

The chloroplast genome of *A. theophrasti* exhibits a typical tetrad structure, with a length of 160,440 bp. It comprises two identical inverted repeat regions (IRa and IRb, each 25,604 bp), a large single-copy (LSC) region of 89,084 bp, and a small single-copy (SSC) region of 20,148 bp (Figure 1, Table 1). Base composition analysis showed that the contents of A, C, G, and T in the chloroplast genome of *A. theophrasti* were 31.19%, 18.66%, 18.23%, and 31.92%, respectively. The total GC content in the genome is 36.89%, with IRa and IRb regions (both 42.96%) showing higher GC content than the LSC (34.64%) and SSC (31.38%) (Table 1).

### 2.2. Functional Annotation of A. theophrasti M. Chloroplast Genes

A total of 130 genes were annotated in the chloroplast genome of *A. theophrasti*, including 85 mRNA genes, 37 tRNA genes, and 8 rRNA genes with no pseudogenes identified. These genes primarily function in photosynthesis, self-replication, and auxiliary metabolism, such as protein processing and membrane structure maintenance. The functions of some genes remain uncharacterized (Table 2). Regarding gene copy numbers: 73 mRNAs and 23 tRNAs are present as single copies; 6 mRNAs, 7 tRNAs, and 4 rRNAs are duplicated. Intron analysis showed that 11 mRNAs and 8 tRNAs contain one intron, while 4 mRNAs contain two introns (Table 2).

### 2.3. Codon Usage Bias Analysis

Excluding stop codons, the chloroplast genome of *A. theophrasti* contains 20,772 codons encoding amino acids. Leucine (Leu) is the most frequently used amino acid (2408 codons), followed by isoleucine (Ile, 1978 codons) and serine (Ser, 1697 codons).

Further analysis of relative synonymous codon usage degree (RSCU) shows 31 codons with RSCU > 1, among which 29 codons end with A or U. There are 33 codons with RSCU < 1, and 30 codons ending in G or C. Notably, tryptophan (Trp) is encoded by a single codon (UGG) with a RSCU value of 1. Among all codons, the methionine (Met) codon AUG has the highest RSCU value (6.9867), followed by the leucine (Leu) UUA (1.9884) and alanine (Ala) GCU (1.7776) codons. The methionine (Met) codon GUG has the lowest RSCU value (0.0133) (Table 3). 

**Figure 2:**
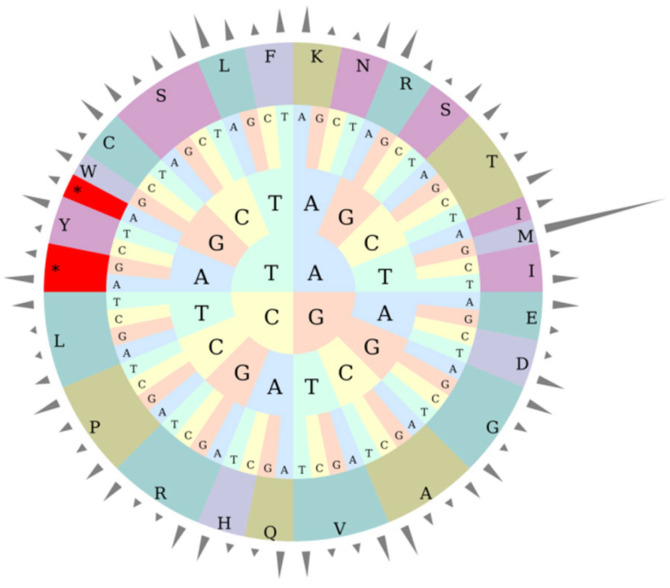
Circular diagram of relative synonymous codon usage (RSCU) in the chloroplast genome of *A. theophrasti*. Note: The outermost cylinder represents the RSCU value, the middle layer consists of amino acids, and the innermost three layers represent codons. Different colors represent different amino acids (abbreviated on the outer ring; for example, A represents alanine, S represents phenylserine, etc.). The letters inside (A, T, C, G) represent nucleotides; asterisks (*) indicate codons with distinctive features.

Combined with the codon circular diagram, it visually displays the distribution of codons corresponding to each amino acid, providing insights into codon usage preferences in the chloroplast genome of A. *theophrasti* (Figure 2).

### 2.4. Repeated Sequence Analysis

**Figure 3:**
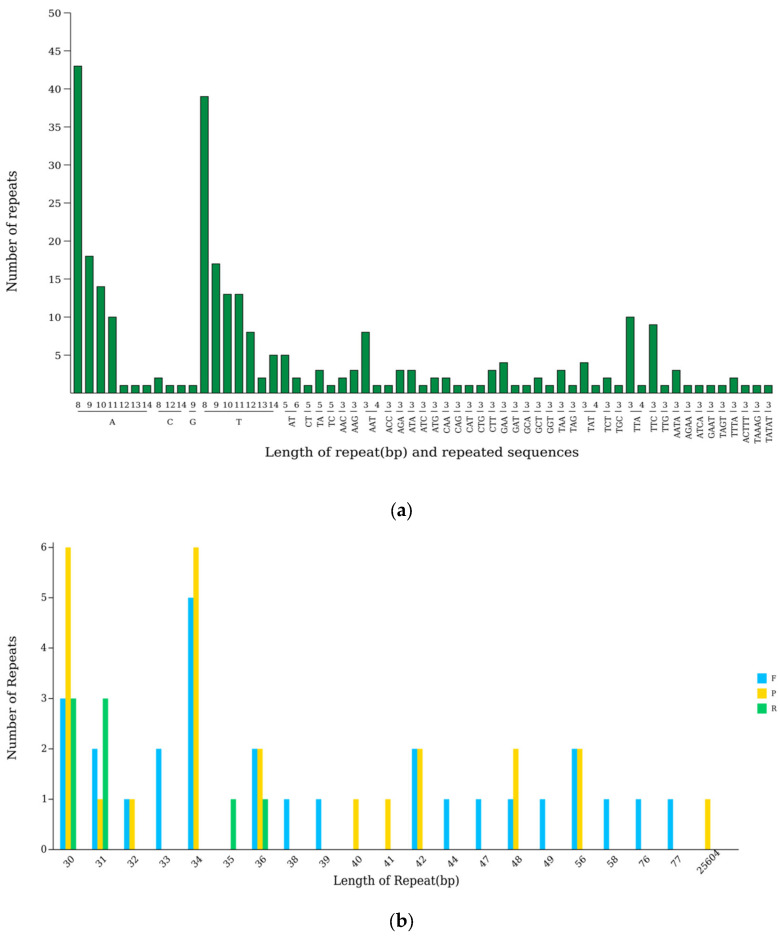
Repeated sequence analysis of the chloroplast genome of *A. theophrasti*. (**a**) Scattered and simple sequence repeats in the chloroplast genome. Note: The horizontal axis represents SSR repeat units, and the vertical axis represents the number of repeat units. (**b**) Length distribution of repeats in the chloroplast genome.

The *A. theophrasti* chloroplast genome contains 61 scattered repeat sequences, including 28 forward (F), 25 palindromic (P), 8 reverse (R), and 0 complementary (C) sequences. Among them, the lengths of most scattered repeat sequences are distributed in the range of 30 to 77 bp, with 30 bp repeats being the most common (12 repeats), followed by 34 bp repeats (11 copies). In addition, one dispersed repeat of 25,604 bp was identified (Figure 3a).

Simple repeat sequences (SSRs) are short tandem repeats of 1 to 6 nucleotides. A total of 288 SSRs were detected in the *A. theophrasti* chloroplast genome, distributed as follows: 205 in LSC regions, 43 in SSC regions, and 40 in the IR regions. From the perspective of the genetic components in different regions. There are 37 SSRs in exons, 41 in introns, and 127 in Intergenic regions in the LSC regions; the number of SSRs in exons, introns, and gene spacers was 23, 1, and 19 in the SSC regions; the number of SSRs in exons, introns, and gene spacers is 17, 8, and 15 in the IR regions. Among these SSRs, the single-nucleotide repeat types are the most abundant. The number of A repeats ranges from 8 to 14, and the quantity is between 1 and 43. The number of T repetitions also ranges from 8 to 14, and the quantity is between 2 and 39. There are a certain number of dinucleotide repeats, such as AT/TA, and numerous types of trinucleotide repeats, such as TTA/TTC, that exist. There are also small amounts of tetranucleotide repeats, pentanucleotide repeats, and hexanucleotide repeats present.

Among the 288 SSRs, the top three most frequent types are A (8 repeats, 14.93%, 43 copies), T (8 repeats, 13.54%, 39 copies), and T (9 repeats, 5.90%, 18 copies) (Figure 3b).

### 2.5. Nucleic Acid Diversity and Boundary Analysis

**Figure 4:**
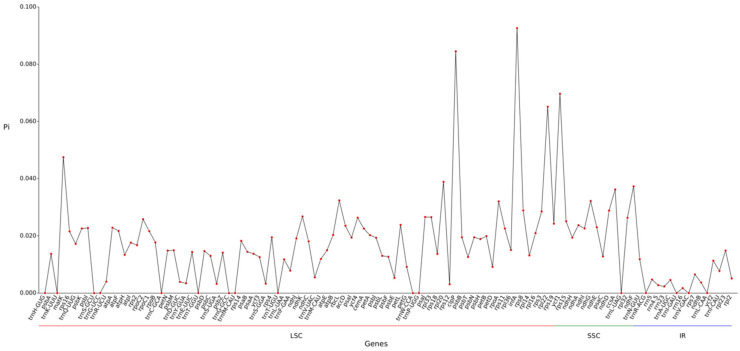
Line chart of gene Pi values.

Nucleotide diversity (Pi) analysis of 113 gene regions in the chloroplast genome of *A. theophrasti* yielded an average Pi value of 0.0170 (Figure 4). Regional Pi values varied as follows: SSC region (0.0275) > LSC region (0.0177) > IR regions (0.0048), indicating higher conservation in the IR regions. Thirty-seven highly variable regions (Pi ≥ 0.02) were identified: 27 in the LSC region 27 l [e.g., infA (0.0926), clpP (0.0845), rpl22 (0.0651)] and 10 in the SSC region [e.g., ycf1 (0.0697), ccsA (0.0362), ndhF (0.0373)]. The most variable locus is infA (0.0926) in the LSC region. 

**Figure 5:**
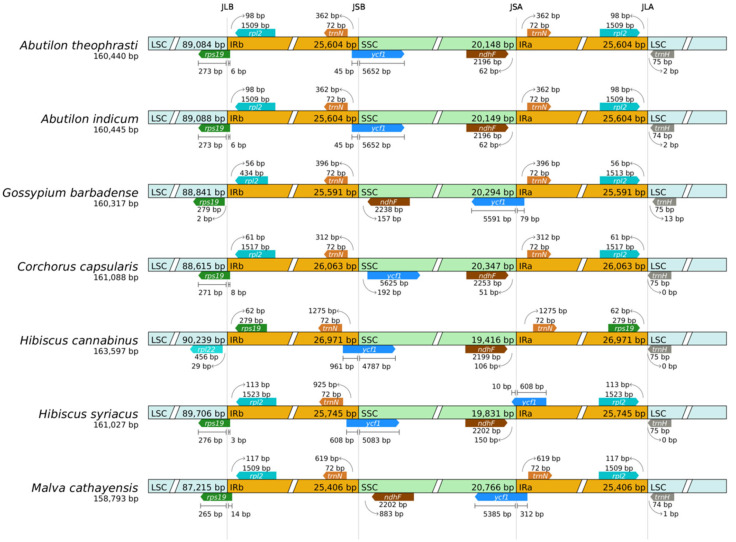
IR/SC boundary analysis.

Expansion and contraction of the IR boundaries are the key factors driving chloroplast genome size variation. The boundary analysis results indicated that the chloroplast genomes of six *Malvaceae* plants and one *Tiliaceae* plant revealed four conserved boundaries: JLB (LSC/IRb), JSB (IRb/SSC), JSA (SSC/IRa), and JLA (IRa/LSC) (Figure 5). Genes near these boundaries include *rps19*, *rpl2*, *rpl22*, *ycf1, ndhF*, *trnN*, and *trnH*. In *Malvaceae* plants, the JLB boundary is located within the *rps19* coding region in all species except *G. barbadense* and *H. cannabinus*. The chloroplast genome of *H*. *cannabinus* differs by 327–343 bp from the other five species, while the five species differ among themselves by 6–16 bp. The JSB boundary is located within the *ycf1* gene region in all species except *G. barbadense* and *M. cathayensis*. A small segment (45–961 bp) of the *ycf1* is in IRb, with the majority (4787–5652 bp) in SSC. For *G*. *barbadense* and *M*. *cathayensis*, the JSB boundary lies between *trnN* and *ndhF*, 396 bp and 619 bp from *trnN* in IRb, respectively. The JSA boundary is located between *ndhF* and *trnN* except for *G*. *barbadense, H*. *syriacus*, and *M. cathayensis*. The *ndhF* gene coding regions in SSC are 62 bp, 62 bp, and 106 bp for these three exceptions, respectively. For *H. syriacus* and *M. cathayensis*, the JSA boundary is within ycf1: 10 bp in SSC and 608 bp in IRa (*H. syriacus*); 5385 bp in SSC and 312 bp in IRa (*M. cathayensis*). The JLA boundary is located 0–13 bp to the left of the trnH coding region in LSC across all species.

For *Corchorus capsularis* (*Tiliaceae*), the JLB boundary is located within *rps19*, differing by 6–16 bp from most species except *H. cannabinus*. The boundary of JSB is between *trnN* and *ycf1*, with greater positional differences from other species. The JSA boundary is located between *ndhF* and *trnN*, differing by 11 to 55 bp from *A. theophrasti*, *A. indicum*, and *H. cannabinus*. The JLA boundary is 0 bp left of *trnH* in LSC. In summary, chloroplast genomes of *Abutilon* species are highly conserved and stable, with consistent IR boundary patterns (e.g., *rps19* crossing the LSC/IRb and ycf1 crossing the IRb/SSC). These patterns serve as a unique phylogenetic signal for distinguishing genera.

### 2.6. Nucleic Acid Diversity Pi Analysis

**Figure 6:**
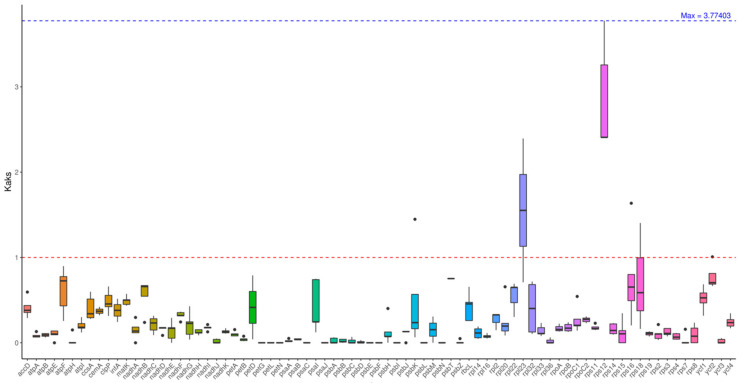
Ka/Ks analysis. Note: The horizontal axis represents gene names, while the vertical axis denotes Ka/Ks ratios. In the box plot, the upper and lower endpoints of the vertical lines above and below the rectangle indicate the upper and lower bounds of the data, respectively. The thick line within the rectangle represents the median, while the upper and lower edges of the rectangle denote the upper and lower quartiles. Data points extending beyond the upper and lower bounds of the rectangle are considered outliers. The red dotted line indicates that the Ka/Ks ratio is equal to 1.

Ka/Ks analysis of chloroplast genes between *A. theophrasti* M. and six related species showed an overall average ratio of 0.24 (Figure 6). Most genes (e.g., *atpA, psaA,* etc.) had Ka/Ks < 1, indicating purifying selection and functional conservation. Ten genes, *atpH*, *petG*, *petL*, *petN*, *psaC*, *psaJ*, *psbF*, *psbI*, *psbL*, and *psbN*, had Ka/Ks = 0, reflecting extreme conservation. Among the highly variable genes, *rps12* (vs. AP009123) had the highest Ka/Ks ratio (3.77); *rpl23* (vs. MK251464) also had Ka/Ks > 1, indicating positive selection. Interfamily comparisons showed a slightly higher Ka/Ks ratio than intrafamily comparisons (*Malvaceae* family only), consistent with phylogenetic differentiation.

### 2.7. Phylogenetic Analysis

**Figure 7:**
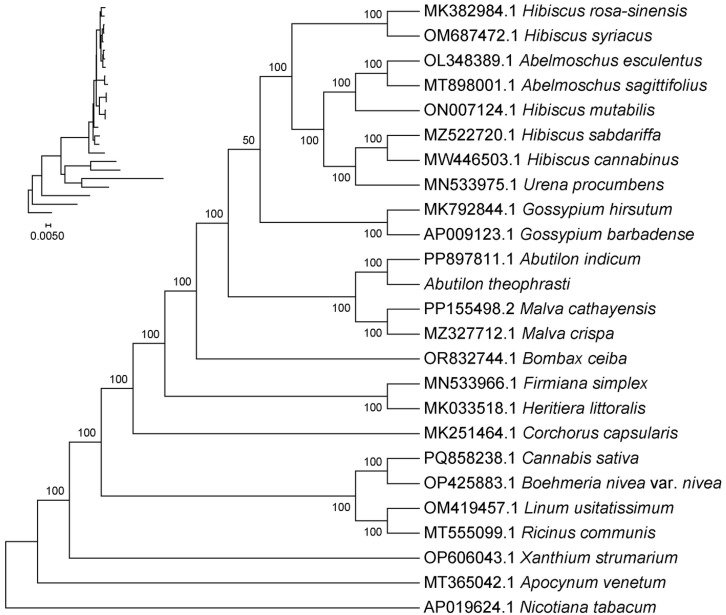
Phylogenetic trees are constructed based on chloroplast genome sequences.

To clarify evolutionary relationships among *Malvaleae* plants, chloroplast genomic data from 24 species were downloaded from the NCBI. On this basis, *N*. *tabacum* of the *Solanaceae* family in the *Solanales* order was used as an exophyte to construct a phylogenetic tree. The results showed that *A*. *theophrasti* is most closely related to its congener *A*. *indicum,* forming a sister group followed by *M*. *cathayensis*, *M. crispa,* and other *Malva* plants, followed by *G*. *hirsutum* and *G*. *barbadense*. *H*. *syriacus* and *H*. *rosa-sinensis*, both belonging to the *Malvaceae* family, are more distantly related to *A*. *theophrasti*. *N*. *tabacum*, as an outgroup, is the most distant from *A*. *theophrasti* (Figure 7).

## 3. Discussion

Chloroplasts are semi-autonomous organelles in the plant genetic system, with a more conserved gene number, composition and arrangement than mitochondrial and nuclear genomes [18]. The chloroplast genome of *A. theophrasti* assembled in this study (160,440 bp, GC content 36.89%) presents a typical tetrameric structure, which is consistent with most terrestrial plants. Comparison with previously published chloroplast genomes of *A. theophrasti* [19,20] shows high consistency in length (160,331 and 160,446 bp) and GC content (36.9%), confirming species-specific conservation. However, the number of annotated genes in our study (130) differs from those of Lv et al. [19] (76) and Yu et al. [20] (113), likely due to variations in annotation criteria, assembly completeness, or potential intraspecific differences. Our analysis, which includes a more comprehensive examination of repetitive sequences, nucleotide diversity, IR boundary dynamics, and genome-wide selective pressure (Ka/Ks), provides an expanded resource for the genomic characterization of *A. theophrasti*. The following discussion integrates these prior findings to contextualize our results within a broader framework. 

Potentially reflecting varietal differences. Our annotation of 130 genes suggests a more complete or differentially interpreted gene set, particularly regarding the identification of distinct transcriptional units and intron-containing genes. These differences underscore the impact of annotation pipelines on comparative genomics and highlight the need for standardized methods. Nevertheless, the core structure—a quadripartite organization with highly conserved LSC, SSC, and IR regions—remains unchanged across all studies, aligning with the characteristic stability of malvaceous chloroplast genomes noted in broader comparative studies [21].

Of the 130 annotated genes, 45 are involved in photosynthesis, highlighting chloroplasts’ central role in this process. Codon usage bias analysis showed leucine, isoleucine, and serine to be the most frequent amino acids. Relative synonymous codon usage (RSCU) is defined by comparing the actual occurrence frequency of a specific codon with its theoretical expected frequency. It is an effective tool for evaluating codon preference. An RSCU greater than 1 indicates a clear preference for the use of that codon [22]. In the chloroplast genome of *A. theophrasti*, there are 31 codons with RSCU greater than 1, among which 93.55% end with A or U. Similar phenomena are widespread in the chloroplast genomes of angiosperms [23,24]. SSRs are widely used for constructing genetic linkage maps, population genetic analysis, and so on. This study found that there were 61 dispersed repeat sequences and 288 SSRs in the chloroplast genome of *A. theophrasti*, which is consistent with winter rapeseed [25], with conserved repeat types such as forward repeats and single-nucleotide repeats. Overall, it shows a relatively high degree of conservatism. This difference reflects the differentiation in evolutionary strategies between the nuclear genome and the chloroplast genome: the nuclear genome enhances chromatin stability through satellite DNA amplification to adapt to environmental stress, while the chloroplast genome retains conserved repeat sequences to maintain the stability of photosynthetic function. It can provide potential candidate molecular markers for the study of the genetic diversity of the *A.* crops. Nucleic acid diversity Pi is an important indicator for measuring the degree of genetic variation within a population. 

A high Pi value indicates rich genetic diversity within the population and can provide potential molecular markers for population genetics [26]. The average nucleic acid diversity (Pi) of all 113 gene regions detected in this study was 0.0170. The average nucleic acid diversity of different regions from largest to smallest was SSC (0.0275), LSC (0.0177), and IR (0.0048), indicating that IR was more conserved compared to the other two regions. Among them, the top four sites with the highest nucleic acid diversity are *infA*, *clpP*, and *rpl22* in the LSC region, and *ycf1* in the SSC region in sequence. These highly variable sites can be used as molecular markers for species identification in the *Malvaceae* family. During the evolution of plant genomes, the expansion or contraction of the IR region is the main driving force for the structural variation of chloroplast genomes, which can provide molecular evidence for species identification and phylogenetic studies [27].

IR boundary analysis revealed that the differences in IR boundaries were mainly related to the positions of *rps19*, *ycf1*, *ndhF*, *trnH*, and *rpl2*. The chloroplast genomes of plants within the *A.* are generally conserved and highly stable, with highly consistent IR boundary types, especially the patterns of *rps19* crossing the LSC/IRb boundary and ycf1 crossing the IRb/SSC boundary, which are consistent with the conclusions drawn from the study of Lycium species by Zhang et al. [28]. The specificity among different genera is strong. The degree of IR boundary expansion (especially the crossing of the *rps19* gene) of the *Abutilon* is a unique phylogenetic signal, which can be used to distinguish different genera. The expansion and contraction of IR boundaries are recognized as a major evolutionary force shaping chloroplast genome diversity in land plants [23]. Our boundary analysis identified *rps19* and *ycf1* as key genes straddling the LSC/IRb and IRb/SSC junctions, respectively, in *A. theophrasti*. This pattern provides a unique phylogenetic signal for the genus *Abutilon*. Notably, Zhong et al. [21] emphasized that such IR boundary dynamics can lead to gene duplication or truncation, potentially altering gene dosage and function. Therefore, the specific IR configuration we observed in *A. theophrasti* may not merely be a taxonomic marker but could reflect underlying evolutionary mechanisms, such as selection for optimized gene expression or a genomic signature of past hybridization events. Investigating the correlation between these structural variations and phenotypic traits or ecological adaptation presents a promising future direction.

Phylogenetic analysis indicates that *A. theophrasti* M. and *A. indicum* of the same genus form a phylogenetic branch together, that is, they have a direct "sister group" relationship. Secondly, there is another branch of the *Malva* plants, *M. cathayensis* and *Malva crispa*. Most closely related to *A. theophrasti* M. is the *Gossypium*. It indicates that among the plants of the *Malvaceae* family, the *Malvaceae* genus plants are the most evolutionarily close group to the *A.* plants, such as *A. theophrasti* M., followed by the *Gossypium* plants. *H. syriacus* and *H. rosa-sinensis*, both belonging to the *Malvaceae* family, have a relatively distant genetic relationship with *A. theophrasti*. Our phylogenetic reconstruction placed *A. theophrasti* in a clade with its congener *A. indicum*, with members of *Gossypium* as closely related lineages. This contrasts with the findings of Yu et al. [20], who positioned *A. theophrasti* at a basal node within the Malveae tribe. Such topological discrepancies are not uncommon in phylogenetic studies and may arise from several factors: (1) the selection of different outgroups and taxon sampling, which can affect root placement; (2) the choice of phylogenetic inference methods and genomic data partitions (e.g., whole genome vs. coding sequences only); and (3) the potential for incomplete lineage sorting or hybridization history within Malveae, which can confuse phylogenetic signals. Despite this difference, both studies confirm the monophyly of Malvaceae and support clear distinctions among major lineages like *Malva*, *Gossypium*, and *Hibiscus*. Resolving the precise phylogenetic position of *Abutilon* may require a phylogenomic approach incorporating data from both chloroplast and nuclear genomes across a denser sampling of the genus.

## 4. Materials and Methods

### 4.1. Sample and Sequencing

The experimental material was *A. theophrasti* cultivated at the Zhejiang Institute of Landscape Plants and Flowers (Zhejiang Xiaoshan Cotton and Bast Fiber Crops Research Institute, Hangzhou, China) (30°07′ N, 120°23′ E). The young leaves of healthy plants were taken, wrapped in tinfoil, and quickly frozen in liquid nitrogen. After being taken out, they were stored in a −80 °C refrigerator. Total DNA of *A. theophrasti* M. was extracted using the universal plant DNA extraction kit (D312) and sequenced using the Illumina NovaSeq 6000 platform.

### 4.2. Chloroplast Genome Assembly and Functional Annotation

Clean data were obtained by filtering the original data using fastp v0.23.4 software [29]. Chloroplast genomes were assembled using GetOrganelle v1.7.7.1 software. The CDS of chloroplasts were annotated respectively by Prodigal v2.6.3 [30], hmmer v3.1b2 [31], and Aragorn v1.2.38 [32]. Predict rRNA and predict tRNA. The chloroplast genome map was drawn using OGDRAW v1.3.1 [33] software.

### 4.3. Analysis of Dispersed and Simple Sequence Repeats

The repetitive sequences were identified by using the vmatch v2.3.09 software [34], and the relevant parameters were set as follows: The Hamming distance is 3, the minimum length is 30 bp, and the identification forms are four types: forward, palindromic, reverse, and complement. Simple sequence repeats (SSRs) were analyzed through MISA v1.0 software [35]. The parameter configuration is as follows: the single-base repetition should be not less than 8 times, the double-base repetition not less than 5 times, and the 3-base, 4-base, 5-base, and 6-base repetitions should occur at least 3 times.

### 4.4. Chloroplast Genomic Nucleic Acid Diversity and Boundary Analysis

Chloroplast genomes of six species of *Malvaceae* and *Tiliaceae* plants were downloaded from NCBI, including *A. indicum* (PP897811.1), *G. barbadense* (AP009123.1), *Corchorus capsularis* (MK251464.1), *H. cannabinus* (MW446503.1), *H. syriacus* (OM687472.1), and *M. cathayensis* (PP155498.2); Global alignment and analysis of homologous gene sequences among different species were conducted using MAFFT v7.427 (--auto mode) software, and nucleic acid diversity (nucleic acid diversity, Pi value) was calculated using the dnasp5 tool [36]. Using sets of Shen cloud platform tools CPJSdraw (http://cloud.genepioneer.com:9929/#/tool/alltool/detail/296, accessed on 15 October 2025), each species chloroplast genome boundary information was subjected to visualization processing. Genomic alignment was performed using the default parameters of Mauve (v2.3.1) software [34].

### 4.5. System Evolution Analysis

Chloroplast genomic data of 24 plant species were downloaded from the NCBI database. It includes chloroplast genomic data of 16 species from the *Malvaceae* family, 1 species from the *Tiliaceae* family, 1 species from the *Cannabaceae* family, 1 species from the *Urticaceae* family, 1 species from the Linaceae family, 1 species from the *Euphorbiaceae* family, 1 species from the *Asteraceae* family, 1 species from the Apocynaceae family, and 1 species from the *Solanaceae* family. *N. tabacum* of the *Solanaceae* family was selected as the exophyte group. This is used to conduct an analysis of phylogenetic relationships. Multiple sequence alignment and evolutionary tree construction were performed, respectively, using MAFFT v7.427 (--auto mode) and RAxML v8.2.10 [37].

## 5. Conclusions

This study clarified the basic characteristics of the chloroplast genome of *A. theophrasti*: it is A typical tetrameric structure, with a total genome length of 160,440 bp, GC content of 36.89%, 130 annotated genes, and its genome codons mostly end with A/U. There are a total of 61 scattered repeat sequences and 288 SSRs. IR boundary analysis revealed that the chloroplast genomes of plants within the genus *A.* are generally conserved, highly stable, and have strong specificity among different genera, which are their unique phylogenetic signals and can be used to distinguish different genera. Phylogenetic analysis shows that *A. indicum* is most closely related to *A. theophrasti*. It has a relatively close evolutionary distance from the *Malva*, providing molecular evidence for the classification and evolutionary research of the *Malvaceae* family. This study clarified the conserved characteristics and evolutionary laws of the chloroplast genome of *A. theophrasti*, providing theoretical support for the phylogenetic research of *A. theophrasti* and other *Malvaceae* plants and the development and utilization of germplasm resources.

## Figures and Tables

**Table 1:** Chloroplast genomic characteristics of *A. theophrasti*.

Region	A Content/%	C Content/%	G Content/%	T Content/%	GC Content/%	Base Length/bp
LSC	31.98	17.87	16.77	33.38	34.64	89,084
SSC	34.54	14.96	16.42	34.08	31.38	20,148
IRa	28.53	22.29	20.67	28.52	42.96	25,604
IRb	28.52	20.67	22.29	28.53	42.96	25,604
Total volume	31.19	18.66	18.23	31.92	36.89	160,440

**Table 2:** Gene annotation of the chloroplast genome of *A. theophrasti* M.

Category	Gene Group	Gene Name
Photosynthesis	Subunits of photosystem I	*psaA*, *psaB*, *psaC*, *psaI*, *psaJ*
	Subunits of photosystem II	*psbA*, *psbB*, *psbC*, *psbD*, *psbE*, *psbF*, *psbH*, *psbI*, *psbJ*, *psbK*, *psbL*, *psbM*, *psbN*, *psbT*, *psbZ*
	Subunits of NADH dehydrogenase	*ndhA* *, *ndhB* *(2), *ndhC*, *ndhD*, *ndhE*, *ndhF*, *ndhG*, *ndhH*, *ndhI*, *ndhJ*, *ndhK*
	Subunits of the cytochrome b/f complex	*petA*, *petB* *, *petD* *, *petG*, *petL*, *petN*
	Subunits of ATP synthase	*atpA*, *atpB*, *atpE*, *atpF* *, *atpH*, *atpI*
	Large subunit of Rubisco	*rbcL*
	Subunits photochlorophyllide reductase	*-*
Self-replication	Proteins of the large ribosomal subunit	*rpl14*, *rpl16* *, *rpl2* *(2), *rpl20*, *rpl22*, *rpl23(2)*, *rpl32*, *rpl33*, *rpl36*
	Proteins of the small ribosomal subunit	*rps11*, *rps12* **(2), *rps14*, *rps15*, *rps16* *, *rps18*, *rps19*, *rps2*, *rps3*, *rps4*, *rps7(2)*, *rps8*
	Subunits of RNA polymerase	*rpoA*, *rpoB*, *rpoC1* *, *rpoC2*
	Ribosomal RNAs	*rrn16(2)*, *rrn23(2)*, *rrn4.5(2)*, *rrn5(2)*
	Transfer RNAs	*trnA-UGC* *(2), *trnC-GCA*, *trnD-GUC*, *trnE-UUC*, *trnE-UUC* *(2), *trnF-GAA*, *trnG-GCC*, *trnG-UCC* *, *trnH-GUG*, *trnI-CAU(2)*, *trnK-UUU* *, *trnL-CAA(2)*, *trnL-UAA* *, *trnL-UAG*, *trnM-CAU*, *trnN-GUU(2)*, *trnP-UGG*, *trnQ-UUG*, *trnR-ACG(2)*, *trnR-UCU*, *trnS-GCU*, *trnS-GGA*, *trnS-UGA*, *trnT-GGU*, *trnT-UGU*, *trnV-GAC(2)*, *trnV-UAC* *, *trnW-CCA*, *trnY-GUA*, *trnfM-CAU*
Other genes	Maturase	*matK*
	Protease	*clpP* **
	Envelope membrane protein	*cemA*
	Acetyl-CoA carboxylase	*accD*
	c-type cytochrome synthesis gene	*ccsA*
	Translation initiation factor	*infA*
	other	*-*
Genes of unknown function	Conserved hypothetical chloroplast ORF	*ycf1*, *ycf2(2)*, *ycf3* **, *ycf4*

Note: Gene *: Contains one intron; Gene **: Contains two introns; Gene: Pseudogene; Gene (2): Gene with copy number greater than 1, with copy number indicated in parentheses.

**Table 3:** Relative synonymous codon usage analysis of *A. theophrasti*.

Amino Acid	Codon	Count	RSCU	Amino Acid	Codon	Count	RSCU	Amino Acid	Codon	Count	RSCU
Ter	UAA	45	1.7088	Ile	AUA	620	0.9402	Arg	AGA	395	1.7478
Ter	UAG	17	0.6456	Ile	AUC	374	0.5673	Arg	AGG	144	0.6372
Ter	UGA	17	0.6456	Ile	AUU	984	1.4925	Arg	CGA	315	1.3938
Ala	GCA	339	1.0464	Lys	AAA	901	1.5142	Arg	CGC	110	0.4866
Ala	GCC	225	0.6944	Lys	AAG	289	0.4858	Arg	CGG	99	0.438
Ala	GCG	156	0.4816	Leu	CUA	331	0.825	Arg	CGU	293	1.2966
Ala	GCU	576	1.7776	Leu	CUC	145	0.3612	Ser	AGC	106	0.375
Cys	UGC	59	0.4738	Leu	CUG	147	0.366	Ser	AGU	346	1.2234
Cys	UGU	190	1.5262	Leu	CUU	496	1.236	Ser	UCA	350	1.2372
Asp	GAC	179	0.3926	Leu	UUA	798	1.9884	Ser	UCC	259	0.9156
Asp	GAU	733	1.6074	Leu	UUG	491	1.2234	Ser	UCG	155	0.5478
Glu	GAA	896	1.5046	Met	AUG	535	6.9867	Ser	UCU	481	1.7004
Glu	GAG	295	0.4954	Met	GUG	1	0.0133	Thr	ACA	345	1.2032
Phe	UUC	422	0.6532	Asn	AAC	251	0.4662	Thr	ACC	222	0.774
Phe	UUU	870	1.3468	Asn	AAU	826	1.5338	Thr	ACG	132	0.4604
Gly	GGA	610	1.5156	Pro	CCA	265	1.124	Thr	ACU	448	1.5624
Gly	GGC	176	0.4372	Pro	CCC	175	0.7424	Val	GUA	471	1.5132
Gly	GGG	296	0.7356	Pro	CCG	138	0.5852	Val	GUC	148	0.4756
Gly	GGU	528	1.312	Pro	CCU	365	1.5484	Val	GUG	172	0.5528
His	CAC	145	0.5244	Gln	CAA	634	1.552	Val	GUU	454	1.4588
His	CAU	408	1.4756	Gln	CAG	183	0.448	Trp	UGG	403	1
								Tyr	UAC	167	0.393
								Tyr	UAU	683	1.607

## Data Availability

The original contributions presented in this study are included in the article. Further inquiries can be directed to the corresponding authors. All original data (including sequencing reads and annotated genomes) supporting the reported results have been submitted to NCBI GenBank (Submission ID: PX673627).
